# AI to predict extrauterine growth restriction during transitional nutrition of preterm infants: a retrospective study

**DOI:** 10.1038/s41372-025-02445-4

**Published:** 2025-10-13

**Authors:** Valentina Bozzetti, Linda Greta Dui, Emanuela Zannin, Silvia Riccò, Paola Coglianese, Valeria Cavalleri, Lucia Iozzi, Maria Luisa Ventura, Simona Ferrante

**Affiliations:** 1 Fondazione IRCCS San Gerardo dei Tintori, Monza, Italy; 2 Mucosal Immunology and Biology Research Center, Massachusetts General Hospital for Children, Boston, MA USA; 3 Gastroenterology and Digestive Endoscopy Department, IRCCS IRCCS Ospedale San Raffaele, Milan, Italy; 4 Department of Electronics, Information and Bioengineering, Politecnico Di Milano, Milan, Italy; 5 LearnLab, Fondazione IRCCS Istituto Neurologico Carlo Besta, Milan, Italy

**Keywords:** Outcomes research, Predictive markers

## Abstract

**Objective:**

Extrauterine growth restriction (EUGR) affects 30–97% of preterm infants and is associated with poor outcomes. We used machine learning (ML) to assess how clinical and nutritional factors, particularly during the transition from parenteral to enteral nutrition, influence EUGR.

**Study design:**

This retrospective observational study included 1165 patients (46% with EUGR) born below 33 weeks’ gestation or 1500 g. We developed 10 models to predict EUGR combining two sets of features (all and nutritional features only) across five subgroups of patients (all, extremely preterm, very preterm, moderately preterm, small for gestational age).

**Results:**

Model accuracy was 0.71 (F1-score = Recall = AUROC = 0.71, Precision = 0.72) with nutritional features and 0.79 (F1-score = AUROC = 0.79, Precision = 0.80, Recall = 0.79) with all features. Lower EUGR risk was linked to female sex, higher growth velocity, and lipid intake in week one. Influential factors differed by subgroup.

**Conclusion:**

ML models accurately predicted EUGR across preterm subgroups, highlighting the role of early nutritional and clinical variables.

## Introduction

Extrauterine growth restriction (EUGR) refers to inadequate growth during hospitalization [1]. At discharge, 30% to 97% of preterm infants, particularly those born very preterm or with very low birth weight (VLBW), do not achieve the expected growth based on intrauterine growth charts [1–5].

Inadequate growth in the neonatal intensive care unit (NICU) is associated with adverse short-term outcomes, such as sepsis and prolonged dependence on mechanical ventilation. It can also significantly impact neurodevelopment at 18–22 months corrected age in extremely low birth weight infants [3]. Therefore, providing adequate nutrition and ensuring appropriate growth in the NICU is crucial for this high-risk population [6–9].

The nutritional care of preterm infants in the NICU consists of three phases. The first is the parenteral nutrition (PN) phase, during which the infant relies entirely on intravenous nutrients, with only minimal enteral feeding, to prevent the consequences of fasting and support intestinal health and development. The second phase, known as transitional nutrition (TN), involves weaning from PN to enteral nutrition (EN) with a gradual increase in enteral intake. The third phase is the EN, where the infant is fully established on milk feeds without parenteral support.

The TN phase is the most critical [10,11], as numerous factors can interfere with adequate nutrition. Transitioning from full parenteral support to EN requires crucial decisions regarding modifiable nutritional practices, including vascular access, choice of milk, fortification, formula selection, and the amount of tolerated enteral intake. The EN is frequently interrupted due to feeding intolerance (e.g., abdominal distension, regurgitation) or medical conditions affecting feeding tolerance, such as severe sepsis, necrotizing enterocolitis (NEC), or cardiorespiratory instability.

While interruption of EN is necessary in certain circumstances, promoting early EN is beneficial to prevent gut atrophy, stimulate gastrointestinal maturation, enhance feeding tolerance, and reduce the incidence of NEC [12]. It is noteworthy that EN requirements significantly differ from parenteral requirements as intestinal absorption reduces the bioavailability of some nutrients. Unfortunately, international recommendations on nutrient intake refer solely to PN or EN [13–17], with no guidelines on weaning PN while minimizing nutrient delivery disruption.

Recently, the availability of big data in healthcare has enabled advanced statistical and machine learning (ML) methods to conduct large retrospective studies to evaluate optimal diagnostic and therapeutic strategies, predict outcomes, and support clinical decisions [18]. Specifically, ML methods are valuable for analyzing the complex impact of various clinical and nutritional factors affecting postnatal growth in preterm infants [18,19]. Specifically, the NICU is a highly data-rich environment, which is ideal for the application of ML and artificial intelligence in general. These technologies have the potential to revolutionize image interpretation, leverage electronic health record data to predict acute events (e.g., sepsis, NEC, acute kidney injury), prognosticate critical outcomes (e.g., bronchopulmonary dysplasia, retinopathy of prematurity), and uncover patterns in monitored parameters to enhance clinical decision-making [20–23].

ML combined with electronic clinical records and computerized programs for PN prescription has the potential to improve the analysis of the complex impact of various clinical and nutritional factors affecting postnatal growth in preterm infants. Previous studies employing ML to predict postnatal growth failure have focused on longitudinal microbiome [20] or metabolome data [21]. These studies evaluated the effect of timing, amount, and type of milk [20,22] or total parenteral intake [22]. However, the impact of specific nutrient intakes and TN strategy on postnatal growth has not been investigated.

The present study aims to investigate the effect of clinical and nutritional factors, including specific nutrient intakes during TN, on EUGR, leveraging ML tools applied to the electronic health records of preterm infants below 33 weeks of gestation or 1500 g of birth weight admitted to a neonatal intensive care unit from January 2005 to November 2021.

## Methods

### Study design and population

This retrospective observational study includes preterm infants admitted to the Fondazione IRCCS San Gerardo dei Tintori Neonatal Intensive Care Unit between January 2005 and November 2021. The study was conducted in accordance with the Declaration of Helsinki, and the protocol was approved by Comitato Etico Territoriale Lombardia 3 (ID 4281). Written informed consent was waived because the data were anonymized, and no re-identification codes linking the data to clinical record IDs were retained.

We included infants with a gestational age (GA) below 33 weeks and/or with a birth weight below 1500 g, with complete nutritional data. We excluded infants with medical conditions potentially affecting growth, including chromosomal abnormalities, congenital diseases, and major abdominal surgery. To ensure a diverse dataset for ML algorithms, we did not restrict the sample based on power analysis.

### Clinical data collection

**Fig. 1:**
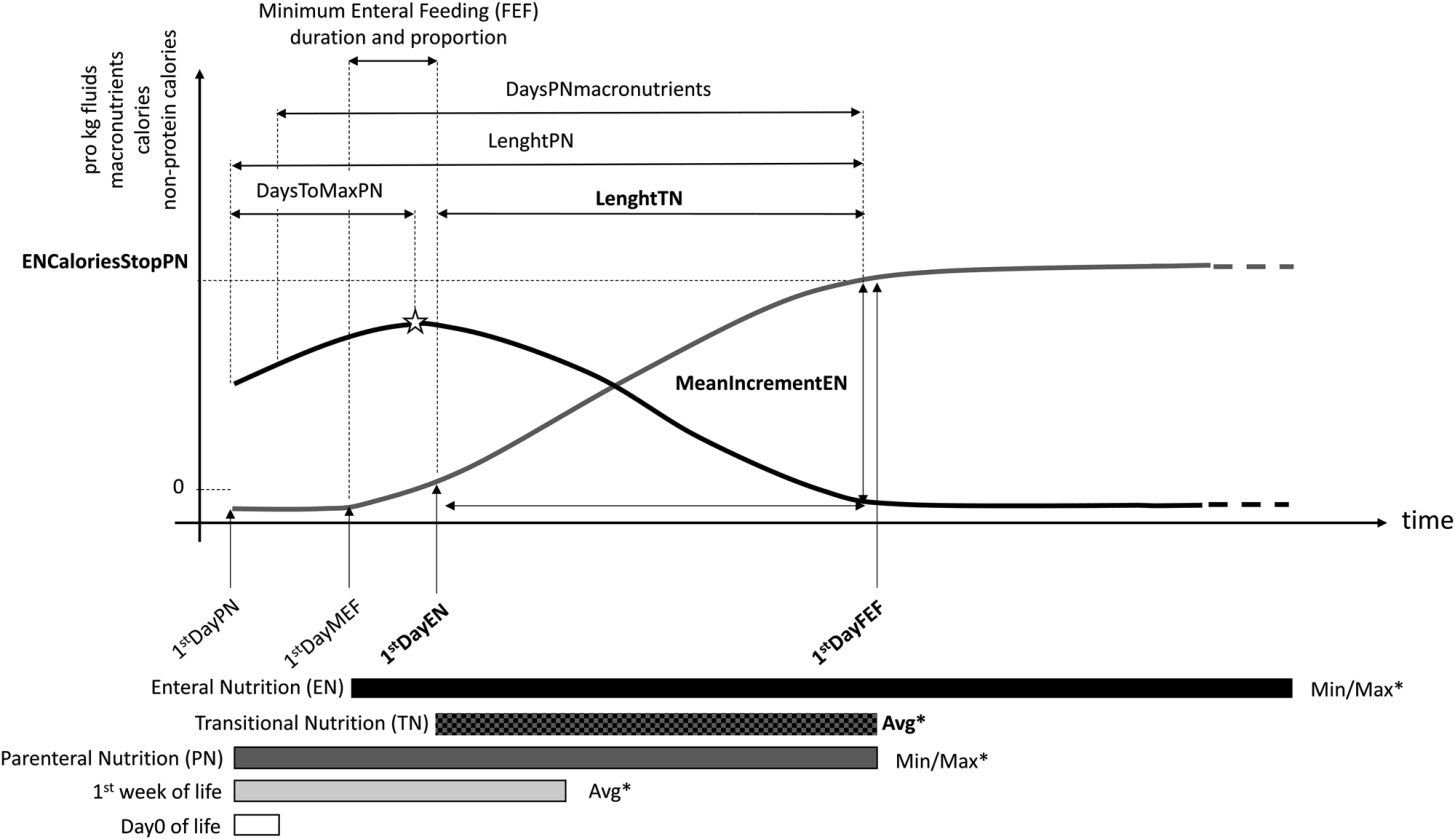
Schematic representation of nutritional features. MeanIncrementEN: Mean daily pro kg increment during TN; DaysToMaxPN: Days to the maximum pro kg amount for a given intake; DaysPNmacronutrients: Days with different parenteral macronutrients; Avg: average amount in g/kg/day, kcal/kg/day or ml/kg/day; Min/Max: maximum/minimum amount in g/kg/day, kcal/kg/day or ml/kg/day; *: computed for PN, EN, and cumulative pro kg intakes of proteins, glucose, lipids, fluids, calories, and non-protein calories.

Data were extracted from the electronic medical records to derive 171 features, including patient demographics, anthropometric measurements, antenatal and perinatal factors, clinical complications, respiratory support needs, antibiotic use, nutritional strategies, and average growth velocity during the first week of life [23] (see eTable 1). Figure 1 provides an overview of the computed nutritional features based on macronutrient (glucose, proteins, and lipids) raw data.

Weight z-scores were calculated using the Intergrowth-21 standards [24], which have demonstrated good concordance with the Fenton reference standard [25]. EUGR was binary coded, considering a loss of at least one weight z-score between birth and the 36th week of postmenstrual age (PMA) or at discharge, whichever occurred first [26].

### Machine learning models development

To assess the impact of the nutritional strategy on postnatal growth, we developed several ML models to predict EUGR using clinical data and parameters describing the nutritional strategy.

Specifically, we built 10 datasets combining 5 groups of patients: (a) all patients, (b) below 28 + 0 weeks’ gestation (extremely preterm, EP), (c) between 28 + 0 and 31 + 6 weeks’ gestation (very preterm, VP), (d) above 32 + 0 weeks (moderate-to-late preterm, MP), and (e) small for gestational age (SGA); and two subsets of features: (1) all features, (2) nutritional features only.

**Fig. 2:**
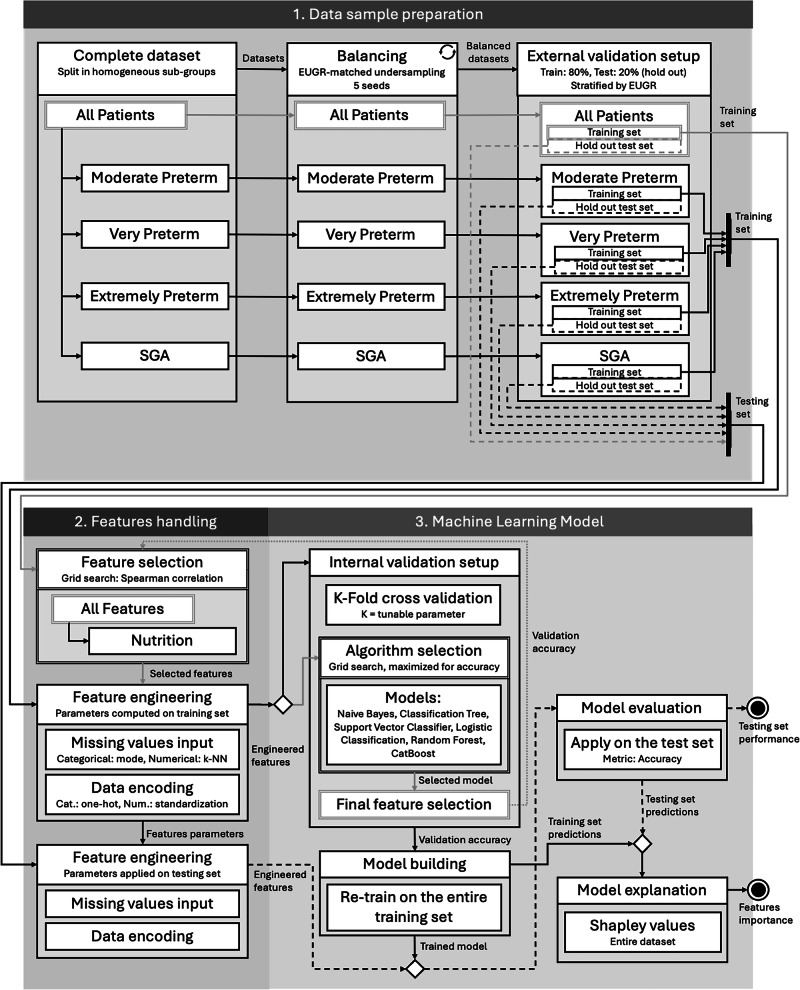
Flowchart of the applied machine learning procedure. The steps marked with double lines are performed on the all-patients all-features dataset only, and applied to the others. Circular arrow: iterations for undersampling (5 seeds).

Figure 2 summarizes the ML models development. First, we performed majority-class undersampling—stratifying by GA for group (a)—to balance the number of EUGR and non-EUGR patients. Next, we split the dataset into training (80%) and validation (20%) sets to assess the internal and external validity of the prediction.

Then, we performed the following steps on the EUGR-balanced training set:

- Correlation-based feature selection [27] on the all-features all-patients dataset (1a), considering different thresholds (*ρ* = 0.7, 0.8, 0.85, 0.9) for feature elimination.
- Standardization and missing values imputation for each of the 5 subsets of patients. Missing weight values were linearly interpolated from the patient’s available data; continuous variables were imputed with 5-Nearest Neighbor; categorical data were imputed with the mode.
- Training of several algorithms (Naïve Bayes, Classification Tree, Random Forest, Support Vector Classifier, Logistic Regression, and CatBoost) on the all-features all-patients dataset (1a) in k-fold cross-validation, considering k a tunable parameter (5 or 10).

The optimal correlation threshold for feature elimination (dataset 1a only—all patients, all features), the best performing model (dataset 1a only), and the number of folds for cross-validation (all datasets) were determined through hyperparameter tuning during cross-validation, optimizing for accuracy.

The best-performing model and configuration were refitted on each of the 10 feature-subgroup combinations.

The following steps were performed on the EUGR-balanced test set:

- Standardization and missing values imputation using parameters computed on the training set to mitigate the risk of bias.
- Fitting of the best-performing model
- Model evaluation and interpretation

The significance of features in predicting EUGR was assessed using Shapley values and expressed as a percentage of importance. The model performance was evaluated in terms of accuracy, F1-score, precision, recall, and area under the receiving operating characteristic curve (AUROC). For each of the 10 feature-subgroup combinations, undersampling, model fitting, and validation were repeated with five random seeds, and performance was averaged. This improvement in robustness included a diverse subset of patients for each repetition. Data were analyzed using Python 3.10.12.

### Statistical analysis

The main descriptors of infants’ condition were expressed as medians and interquartile ranges or numbers and percentages. The significance of differences was evaluated using the Wilcoxon rank sum test for continuous variables and Pearson’s Chi-squared test for dichotomous variables. Alpha values < 0.05 were considered statistically significant. Data were analyzed in R 4.0.1.

## Results

### Study population

One thousand five hundred and fifty-six newborns <33 weeks’ gestation or below 1500 g of birth weight were born at our center between January 2005 and November 2021.

Ninety patients were excluded due to congenital chromosomal abnormalities, congenital diseases, or major abdominal surgery; 301 were excluded because the nutritional data did not include the TN phase until full enteral feeding (FEF) was achieved (Supplementary Fig. 1). Among patients without complete nutritional data, 87 (29%) received only EN, 4 (2%) were still hospitalized when data were extracted, 118 (39%) died before achieving FEF, 76 (25%) were transferred before achieving FEF, and 16 (5%) had data relative to less than 72 h of FEF. 1165 were included in the analysis, 320 were moderate-to-late preterm, 641 very preterm, 204 extremely preterm, and 309 SGA. Of these 1165 patients, 531 (45.6%) developed EUGR (Table 1). The EUGR subgroup showed significant differences from the non-EUGR group, including more males, lower GA and BW, fewer SGA cases, worse Apgar scores, higher early-onset sepsis rates, poorer respiratory outcomes, and longer hospital stays. Regarding nutritional factors, they started EN later after birth, but at a higher PMA, they had a longer TN phase. They also began FEF at a later postnatal age. Although the difference in PMA at FEF initiation was statistically significant, the difference—less than 1 week—was clinically negligible.

**Table 1:** Characteristics of study participants.

	non-EUGR	EUGR	*p*-value
*N*	634	531	
EP, *n* (%)	76 (12%)	128 (24%)	<0.001
VP, *n* (%)	356 (56%)	285 (54%)	0.43
MP, (%)	202 (32%)	118 (22%)	<0.001
Males, *n* (%)	266 (42%)	325 (61%)	<0.001
GA, weeks	31.00 (29.43, 32.29)	29.86 (28.00, 31.71)	<0.001
BW, g	1330 (1040, 1534)	1220 (970, 1500)	0.004
Growth at birth			
SGA, *n* (%)	195 (30.8%)	114 (21.5%)	<0.001
AGA, *n* (%)	425 (67.0%)	391 (73.6%)	0.02
LGA, *n* (%)	14 (2.2%)	26 (4.9%)	0.02
Apgar score			
1 min	7 (5, 8)	7 (5, 8)	<0.001
5 min	9 (8, 9)	8 (7, 9)	<0.001
Sepsis, *n* (%)			
Early onset	192 (30.3%)	298 (56.1%)	<0.001
Late onset	92 (14.5%)	68 (12.8%)	0.45
NEC, *n* (%)			
Medical	19 (3.0%)	23 (4.3%)	0.29
Surgical	3 (0.5%)	-	0.31
Oxygen dependency			
28 days	96 (15.1%)	117 (22.0%)	0.003
36 weeks PMA	49 (7.7%)	59 (11.1%)	0.06
IVH, *n* (%)	8 (1.3%)	18 (3.4%)	0.02
ROP, *n* (%)	7 (1.1%)	7 (1.3%)	0.95
Death, *n* (%)	-	2 (0.4%)	0.21
Length of stay, days	32 (23, 50)	46 (28, 66)	<0.001
Nutritional factors			
First day EN	4 (2, 7)	6 (1, 9)	<0.001
Length TN, days	7 (5, 9)	8 (5, 12)	<0.001
First day FEF	11 (7, 17)	14 (9, 22)	<0.001
PMA at FEF, weeks	32.71 (31.57, 33.71)	32.29 (31.00, 33.43)	<0.001
Δ weight z-score	–0.6 (–0.8, –0.3)	–1.4 (–1.8, –1.2)	<0.001

Data are expressed as median (Q1, Q3) unless otherwise stated.

*EUGR* extrauterine growth restriction, *EP* extremely preterm, *VP* very preterm, *MP* moderate-to-late preterm, *GA* gestational age, *BW* birth weight, *SGA* small for gestational age, *AGA* appropriate for gestational age, *LGA* large for gestational age, *NEC* necrotising enterocolitis, *PMA* postmenstrual age, *IVH* intraventricular hemorrhage, *ROP* retinopathy of prematurity, *EN* enteral feeding, *TN* transitional nutrition, *FEF* full enteral feeding, *Δ weight z-score* difference in body weight z-score between birth and 36 weeks’ PMA or discharge, whichever came first.

Surgical NEC and death occurred in three and two cases, respectively, but such proportions do not reflect the prevalence in the overall population. Indeed, of the 301 infants excluded due to lack of TN data and/or because they did not reach FEF, 118 died before 36 weeks of PMA, and 76 were transferred to other hospitals, some for abdominal surgery.

### Best-performing model and configuration

The optimal number of folds for cross-validation was 10 for datasets comprising all patients, MP, and VP; 5 for datasets comprising EP and SGA. We identified an optimal correlation threshold of 0.85 for feature elimination, leading to the selection of 54 features, including 31 nutritional parameters and the average growth velocity over the first week of life growth (see eTable 1). CatBoost was selected as the best-performing model with an accuracy of 0.76 ± 0.05 (mean and standard deviation across validation folds during internal validation) (see eTable 2 for the complete comparison).

### Features importance

**Fig. 3:**
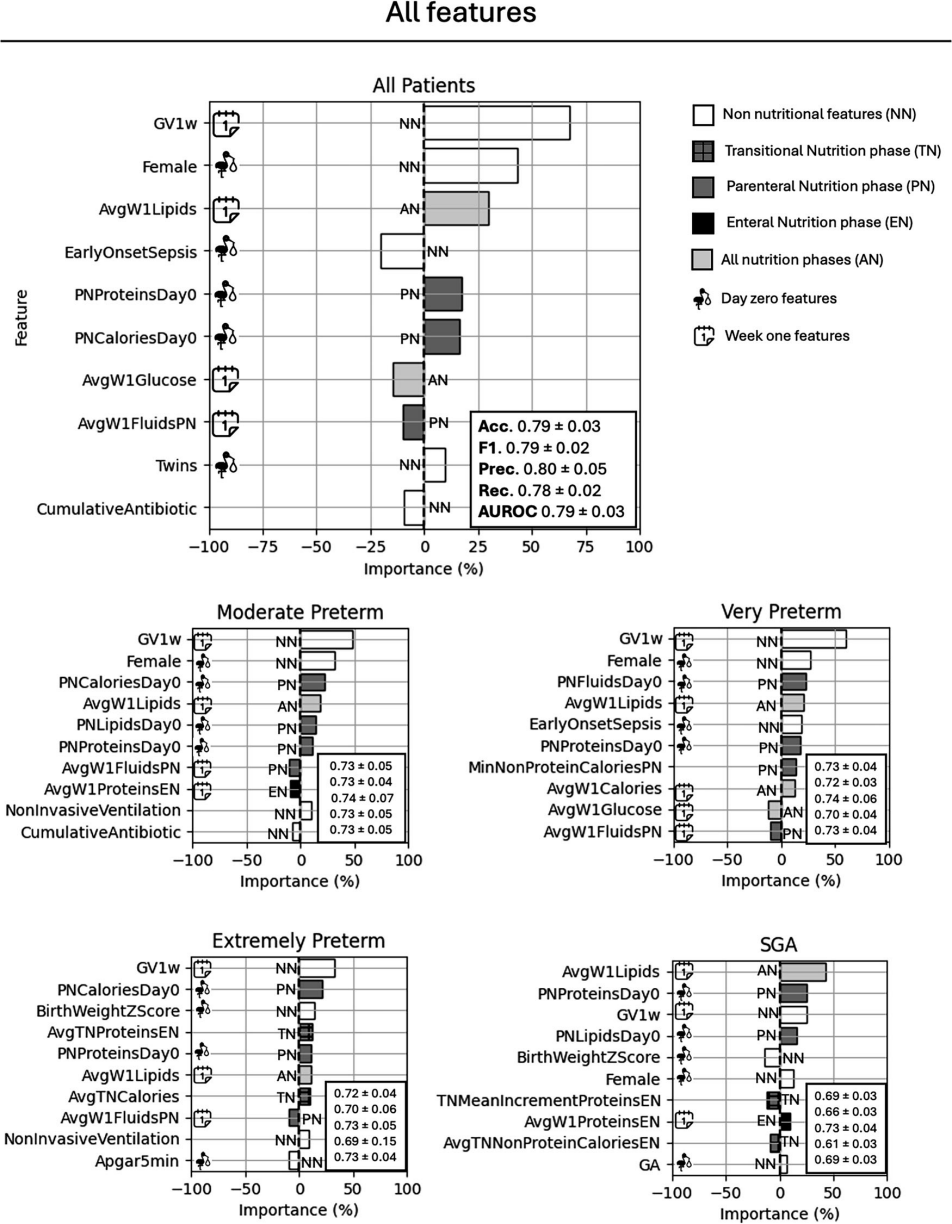
Features importance expressed as percentage impact on the prediction, considering all features. Positive values are associated with better outcomes (decreased risk of EUGR). External performance metrics across the five EUGR-balanced datasets are reported in the boxes. *Acc* accuracy, *F1* F1-score, *Prec* precision, *Rec* recall, *AUROC* area under the receiving operating characteristic curve.

**Fig. 4:**
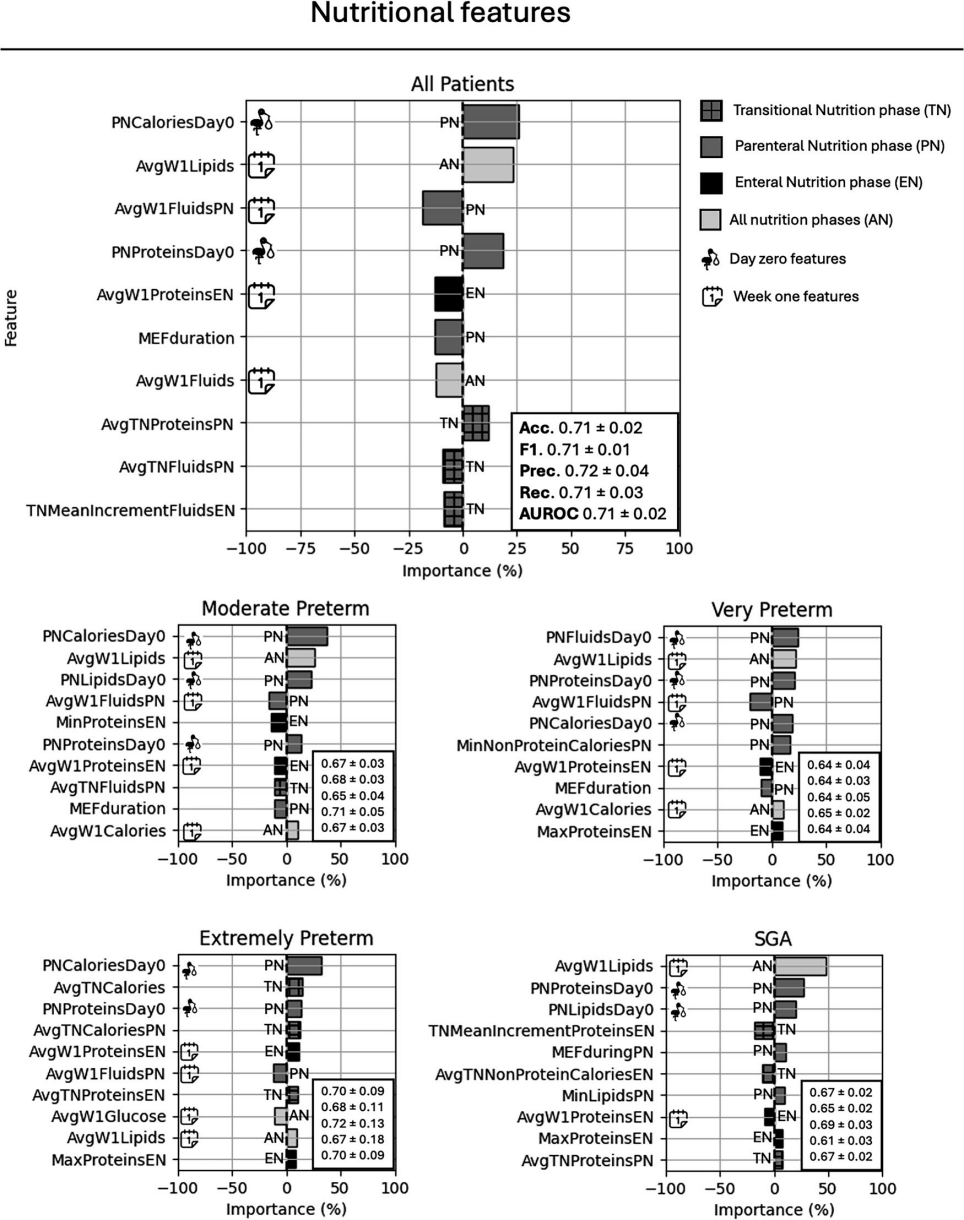
Features importance expressed as percentage impact on the prediction, considering nutritional features only. Positive values are associated with better outcomes (decreased risk of EUGR). External performance metrics across the five EUGR-balanced datasets are reported in the boxes. *Acc* accuracy, *F1* F1-score,* Prec* precision, *Rec* recall, *AUROC* area under the receiving operating characteristic curve.

On the whole population, the model including only nutritional features had an accuracy of 0.71 ± 0.02 (mean and standard deviation across the five EUGR-balanced datasets) in external validation; the model’s performance in terms of F1-score, precision, recall, and AUROC was 0.71 ± 0.01, 0.72 ± 0.04, 0.71 ± 0.03, and 0.71 ± 0.02, respectively. The model with all features had an accuracy of 0.79 ± 0.03, F1-score = 0.79 ± 0.02, Precision = 0.80 ± 0.05, Recall = 0.78 ± 0.02, and AUROC 0.79 ± 0.03. Details on internal and external validation are reported in eTable 3 for one EUGR-balanced dataset and in Figs. 3 and 4 for all the seeds. Figures 3 and 4 show the most important features for EUGR prediction, considering all features and only nutritional features, respectively. Lower growth velocity in the first week of life was among the most important factors associated with EUGR across groups. Lipid intake was associated with better growth, especially in SGA patients. Being male was among the most important predictors of EUGR in the whole population, but it was relatively less important in extremely preterm infants. Regarding TN variables, in the entire population, the most important factor was the average parenteral protein intake (importance = 12.5%), whilst, in the SGA sub-group, it was the mean increment of enteral proteins (importance = 18.4%), with high values associated with increased risk of EUGR. TN features were generally more important in EP and SGA infants than in other subgroups.

## Discussion

We investigated the effect of clinical and nutritional factors on EUGR, focusing on the transition from parenteral to enteral feeding. This study significantly advances understanding of the complex factors contributing to EUGR in preterm infants. Specifically, it provides an innovative and comprehensive analysis of the interplay between clinical and nutritional variables by leveraging ML tools and a robust dataset from over 15 years of electronic health records. Furthermore, the use of explainable artificial intelligence techniques (e.g., Shapley values) offers a transparent interpretation of feature importance, enhancing the clinical relevance of the findings across different patient subgroups.

### Models accuracy

The prediction accuracy was comparable to previous reports [28] and, more importantly, consistent between internal and external validation, demonstrating good generalization capability and robustness of the models. Even if the models which include both clinical and nutritional features achieved the best results—confirming a multifactorial influence on preterm growth—nutritional features alone achieved accuracies that were only slightly below the state of the art, confirming the key role of an adequate nutrition strategy to meet growth goals. The finding that the predictive accuracy improved when integrating nutritional features underscores the importance of early macronutrient management, such as lipid and protein intake, in preventing EUGR.

### Opportunities for an early intervention

Growth velocity during the first week of life consistently affected EUGR regardless of the patient subgroup, indicating an association between slow early growth rates and nutritional deficits [29,30]. Lipid intake during the first week of life was also important across subgroups, highlighting the critical role of early nutritional interventions. Indeed, starting adequate PN within the first hours of life is essential to promote optimal growth and prevent energy deficits in the early postnatal period [31,32]. Interestingly, even if extremely preterm and SGA infants are characterized by long hospital stays (median and quartiles: 79 (63, 100) days and 42 (29, 61) days, respectively), variables related to the first day and the first week of life remain important, as in the other groups.

### Effect of nutrition during the transition phase

An innovative aspect of the present study is the focus on TN. Interestingly, we identified specific factors relative to the TN phase that significantly predicted EUGR. In particular, we observed a positive impact of protein content in PN on growth. In contrast, increased fluid intake, both in PN and EN, negatively impacted growth. These findings suggest that adequate provision of amino acids is more critical for promoting lean mass accretion and supporting growth during the TN phase than fluid provision, as supported by the scientific literature [33]. Such an interpretation is consistent with previous reports. Meio et al. [33] recently analyzed the total energy-to-protein ratio, finding that during the second week of life, typically corresponding to the TN phase, a median ratio below 25 kcal/g of protein was significantly associated with weight-based EUGR [34]. A Cochrane Review comparing higher versus lower parenteral amino acid intake revealed that increased amino acid intake in PN reduced the incidence of postnatal growth failure without affecting mortality [35].

Variables related to the TN phase are among the most important predictors of EUGR in extremely preterm and SGA infants. This finding, together with the longer duration of the TN phase (extremely preterm: 13 (9, 20) days; SGA: 8 (6, 12) days), suggests a higher risk of feeding difficulties and complications during the TN phase in these subgroups of patients, likely due to their smaller size and immature digestive systems [36].

### Effect of specific macronutrients

Another specificity of our study is that we included individual macronutrients in the prediction models, allowing us to speculate on their relative importance in determining adequate postnatal growth. Interestingly, we found that high fluid and glucose intakes are associated with an increased risk of EUGR. The discrepancy between the beneficial effect of lipid intake through PN on growth and the negative impact of high fluid and glucose intake may be explained by the fact that lipid administration can be considered a proxy for complete PN. Our findings are consistent with a meta-analysis that demonstrated a correlation between the early introduction of high lipid intake and growth in preterm infants [26].

### Differential effect of nutritional factors on specific subgroups of patients

The stratification of the study population into homogeneous subgroups had technical and clinical implications. From a technical perspective, increased data homogeneity could benefit ML models, potentially compensating for the reduction in sample size. Such a dual effect is evident from the accuracy results, which remained nearly stable. From a clinical perspective, stratification could help identify differences in the determinants of growth among subgroups of patients and guide tailored corrective actions.

Regarding the EP group, our findings indicate that ensuring adequate parenteral protein intake is essential, especially immediately following birth. During the TN phase, it is crucial to maintain sufficient caloric intake through both parenteral and enteral routes. Additionally, a higher enteral protein intake has a positive impact on growth.

Although a rapid transition might benefit the general preterm population, it may not be appropriate for SGA infants. In these cases, a more cautious advancement of enteral feeding and a gradual reduction of PN is recommended, as a swift increase in enteral protein intake could hinder growth. This suggests that their ability to absorb and utilize amino acids may be impaired. Indeed, a faster increase in enteral caloric intake was associated with a higher risk of EUGR in these infants. Additionally, we observed a positive impact of increased parenteral protein intake on growth in SGA infants. Such a finding aligns with other studies that link poor growth outcomes at discharge to protein deficits during weaning from PN [5,10]. An adequate protein intake is also crucial for brain growth and development, as proteins serve as the primary structural framework of the brain, as suggested by the positive correlation between higher protein intake, increased head circumference growth, and enhanced neurodevelopmental outcomes [37].

### Differential effect of clinical factors on postnatal growth in specific subgroups of patients

Surprisingly, gestational age and birth weight were not among the most important variables for predicting growth outcomes, despite being well-established risk factors for EUGR [38]. Similarly, we observed an unexpected behavior in the role of sex: while males are generally considered at higher risk for EUGR [38], sex becomes less significant in more critical situations, such as in extremely preterm or SGA infants. This finding suggests that, in a multidimensional approach, commonly recognized risk factors are less critical compared to other variables.

### Study limitations

A limitation of this study is that excluding patients who did not complete the TN phase led to the loss of a significant number of infants who were transferred for abdominal surgery and who died (particularly in the extremely preterm subgroup), introducing survivorship bias. However, since our analysis focused specifically on the effect of TN on postnatal growth, we found it reasonable to exclude patients with incomplete data on the intervention of interest. Another limitation is the single-centre study design, which may limit the generalizability of results. In our unit, the electronic clinical report has been customized to receive detailed data from the software used for the computerized prescription of PN and integrate them with the prescription of EN. Such customization allows us to analyze the association of individual nutrients across time on relevant outcomes, but makes it difficult to aggregate our data with other centres. Our results hold value as they may serve as preliminary data for designing multicentric, prospective studies investigating the effect of specific TN strategies on postnatal growth.

## Conclusions

This study is the first to analyze such a comprehensive volume of data, encompassing both nutritional and non-nutritional factors, to assess the risk factors for EUGR using an innovative machine-learning model. The approach not only represents a significant advancement in understanding EUGR but also sets a new standard for predictive modeling in neonatal care.

## Supplementary information

Supplemental material

## Data Availability

The datasets generated during and/or analysed during the current study are not publicly available due to privacy or ethical restrictions, but are available from the corresponding author on reasonable request.

## Code availability

The code for machine learning models is available from the corresponding author on reasonable request.
