# Supplementary material for: AI to predict extrauterine growth restriction during transitional nutrition of preterm infants: a retrospective study
Source: J Perinatol. 2025 Oct 13;46(3):416–24. doi: 10.1038/s41372-025-02445-4 (PMC13008769; doi:10.1038/s41372-025-02445-4)
Supplement: Supplementary file 1 — Supplemental material [file 41372_2025_2445_MOESM1_ESM.docx]

Online-only Supplements for the paper **“****AI to predict growth failure during transitional nutrition of preterm infants”**

1. eFigure 1: Strobe flow chart.
2. eTable 1: Features list.
3. eTable 2: Classification algorithm choice. Mean (± standard deviation) for 10-fold cross-validation. The best result in bold.
4. eTable 3: Cross-validation (i.e., internal validation) and external validation accuracy results from one random split of the dataset.


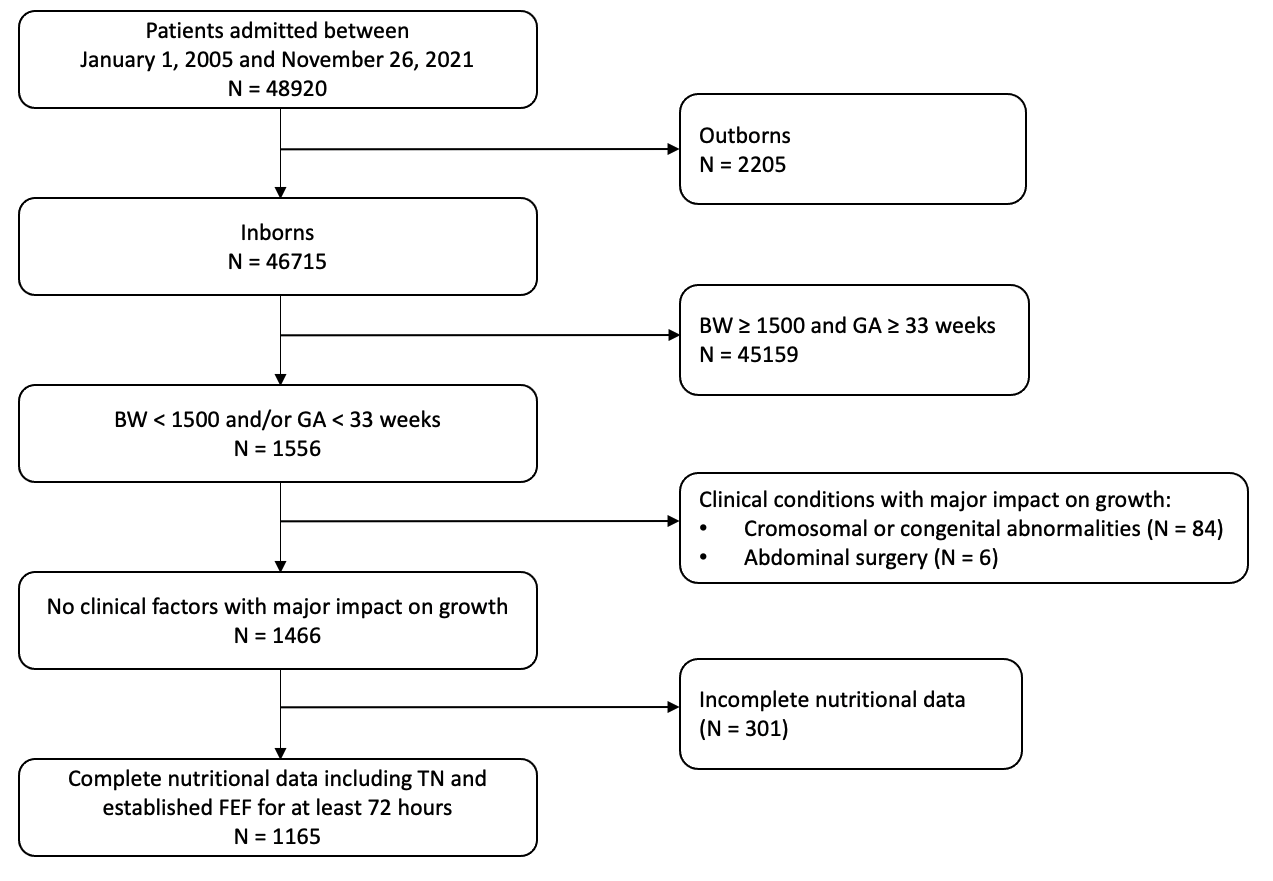


*eFigure 1: Strobe flow chart.*

| **Domain** | **Feature** | **Description** | **Included** | **Missing N (%)** |
| --- | --- | --- | --- | --- |
| Perinatal | GA | Completed weeks of gestation at birth | x | - |
|  | Sex | Binary coded with 1 = female and 0 = male | x | - |
|  | BW | Birth weight in grams | x | - |
|  | BirthWeightZScore | Birth weight z-score, calculated according to the Intergrowth-21 | x | - |
|  | BirthLength | Birth length in centimiters |  | 118 (10.1%) |
|  | HeadCircumference | Head circumference at birth in centimeters |  | 117 (10.0%) |
|  | Apgar1min | Apgar score at 1 minute of life | x | 8 (0.7%) |
|  | Apgar5min | Apgar score at 5 minute of life | x | 3 (0.3%) |
| Prenatal | Twins | Binary coded with 1 = multiple pregnancy and 0 = singleton | x | 32 (2.7%) |
|  | FGR | Fetal growth restriction |  | - |
|  | PROM | Premature rupture of membranes before the onset of labor. Three levels: "No", "< 18 hours", "> 18 hours". | x | - |
|  | Preeclampsia | Binary coded |  | - |
|  | Eclampsia | Binary coded |  | - |
|  | GestationalDiabetes | Binary coded |  | - |
|  | ThyroidPathology | Binary coded |  | - |
|  | PathologicalFetalDoppler | Binary coded |  | - |
|  | MotherAge | Mother's age in years | x | 16 (1.4%) |
|  | Smoke | Indicates whether the mother smoked during pregnancy. Three levels: "No", "< 10 cigarettes/day", "> 10 cigarettes/day". | x | - |
|  | Alcohol | Alcohol use during pregnancy | x | - |
| Nutritional | FirstDayEN | The first of 3 consecutive days with EN fluids > 25 ml/kg/d (day of life) |  | - |
|  | FirstDayFEF | The first of FEF in days |  | - |
|  | FirstDayPN | First day with a parenteral intake in days | x | - |
|  | LengthTN | Duration of TN phase in days | x | - |
|  | LengthPN | Duration of PN in days, from first day with PN to the last day of TN phase |  | - |
|  | FirstDayMEF | First day with MEF | x | 21 (1.8%) |
|  | MEFduration | Number of days from first day of MEF to first day EN | x | 21 (1.8%) |
|  | MEFdays | Number of days of life with MEF |  | 9 (0.8%) |
|  | MEFduringPN | Proportion of days with MEF over days with total PN | x | 9 (0.8%) |
|  | ENCaloriesDay0 | Enteral calories on day the first of life in kcal/kg/day |  | - |
|  | ENNonProteinCaloriesDay0 | Enteral non-protein calories on day the first of life in kcal/kg/day |  | - |
|  | ENProteinCaloriesDay0 | Enteral protein calories on day the first of life in kcal/kg/day |  | - |
|  | ENFluidsDay0 | Enteral fluids amount on day of life 0 in ml/kg/day |  | - |
|  | ENGlucoseDay0 | Enteral glucose amount on day of life 0 in g/kg/day |  | - |
|  | ENLipidsDay0 | Enteral lipids amount on day of life 0 in g/kg/day |  | - |
|  | ENProteinsDay0 | Enteral proteins amount on day of life 0 in g/kg/day |  | - |
|  | PNCaloriesDay0 | Parenteral calories on day the first of life in kcal/kg/day | x | - |
|  | PNNonProteinCaloriesDay0 | Parenteral non-protein calories on day the first of life in kcal/kg/day |  | - |
|  | PNProteinCaloriesDay0 | Parenteral protein calories on day the first of life in kcal/kg/day |  | - |
|  | PNFluidsDay0 | Parenteral fluids amount on day of life 0 in ml/kg/day | x | - |
|  | PNGlucoseDay0 | Parenteral glucose amount on day of life 0 in g/kg/day |  | - |
|  | PNLipidsDay0 | Parenteral lipids amount on day of life 0 in g/kg/day | x | - |
|  | PNProteinsDay0 | Parenteral proteins amount on day of life 0 in g/kg/day | x | - |
|  | CaloriesDay0 | Total calories on day the first of life in kcal/kg/day |  | - |
|  | FluidsDay0 | Total fluids amount on day of life 0 in ml/kg/day |  | - |
|  | GlucoseDay0 | Total glucose amount on day of life 0 in g/kg/day |  | - |
|  | LipidsDay0 | Total lipids amount on day of life 0 in g/kg/day |  | - |
|  | ProteinsDay0 | Total proteins amount on day of life 0 in g/kg/day |  | - |
|  | TNMeanIncrementCaloriesEN | Mean daily pro kg calories increment over TN phase |  | - |
|  | TNMeanIncrementFluidsEN | Mean daily pro kg fluids increment over TN phase | x | - |
|  | TNMeanIncrementGlucoseEN | Mean daily pro kg increment in glucose over TN phase |  | - |
|  | TNMeanIncrementLipidsEN | Mean daily pro kg increment in lipids over TN phase |  | - |
|  | TNMeanIncrementProteinsEN | Mean daily pro kg increment in proteins over TN phase | x | - |
|  | AvgTNCaloriesEN | Average enteral calories during TN in kcal/kg/day |  | - |
|  | AvgTNNonProteinCaloriesEN | Average enteral non-protein calories during TN in kcal/kg/day | x | - |
|  | AvgTNProteinCaloriesEN | Average enteral protein calories during TN in kcal/kg/day |  | - |
|  | AvgTNFluidsEN | Average enteral fluids during TN in ml/kg/day |  | - |
|  | AvgTNGlucoseEN | Average enteral glucose during TN in g/kg/day |  | - |
|  | AvgTNLipidsEN | Average enteral lipids during TN in g/kg/day |  | - |
|  | AvgTNProteinsEN | Average enteral proteins during TN in g/kg/day | x | - |
|  | AvgTNCaloriesPN | Average parenteral calories during TN in kcal/kg/day | x | - |
|  | AvgTNNonProteinCaloriesPN | Average parenteral non-protein calories during TN in kcal/kg/day |  | - |
|  | AvgTNProteinCaloriesPN | Average parenteral protein calories during TN in kcal/kg/day |  | - |
|  | AvgTNFluidsPN | Average parenteral fluids during TN in ml/kg/day | x | - |
|  | AvgTNGlucosePN | Average parenteral glucose during TN in g/kg/day |  | - |
|  | AvgTNLipidsPN | Average parenteral lipids during TN in g/kg/day |  | - |
|  | AvgTNProteinsPN | Average parenteral proteins during TN in g/kg/day | x | - |
|  | AvgTNCalories | Average calories during TN in kcal/kg/day | x | - |
|  | AvgTNNonProteinCalories | Average non-protein calories during TN in kcal/kg/day |  | - |
|  | AvgTNProteinCalories | Average protein calories during TN in kcal/kg/day |  | - |
|  | AvgTNFluids | Average fluids during TN in ml/kg/day | x | - |
|  | AvgTNGlucose | Average parenteral glucose during TN in g/kg/day |  | - |
|  | AvgTNLipids | Average lipids during TN in g/kg/day |  | - |
|  | AvgTNProteins | Average proteins during TN in g/kg/day |  | - |
|  | AvgW1CaloriesEN | Average enteral calories during the first week of life in kcal/kg/day |  | - |
|  | AvgW1NonProteinCaloriesEN | Average enteral non-protein calories during the first week of life in kcal/kg/day |  | - |
|  | AvgW1ProteinCaloriesEN | Average enteral protein calories during the first week of life in kcal/kg/day |  | - |
|  | AvgW1FluidsEN | Average enteral fluids during the first week of life in ml/kg/day |  | - |
|  | AvgW1GlucoseEN | Average enteral glucose during the first week of life in ml/kg/day |  | - |
|  | AvgW1LipidsEN | Average enteral lipids during the first week of life in ml/kg/day |  | - |
|  | AvgW1ProteinsEN | Average enteral proteins during the first week of life in ml/kg/day | x | - |
|  | AvgW1CaloriesPN | Average parenteral calories during the first week of life in kcal/kg/day | x | - |
|  | AvgW1NonProteinCaloriesPN | Average parenteral non-protein calories during the first week of life in kcal/kg/day |  | - |
|  | AvgW1ProteinCaloriesPN | Average parenteral protein calories during the first week of life in kcal/kg/day |  | - |
|  | AvgW1FluidsPN | Average parenteral fluids during the first week of life in ml/kg/day | x | - |
|  | AvgW1GlucosePN | Average parenteral glucose during the first week of life in ml/kg/day |  | - |
|  | AvgW1LipidsPN | Average parenteral lipids during the first week of life in ml/kg/day |  | - |
|  | AvgW1ProteinsPN | Average parenteral proteins during the first week of life in ml/kg/day |  | - |
|  | AvgW1Calories | Average calories during the first week of life in kcal/kg/day | x | - |
|  | AvgW1NonProteinCalories | Average non-protein calories during the first week of life in kcal/kg/day |  | - |
|  | AvgW1ProteinCalories | Average protein calories during the first week of life in kcal/kg/day |  | - |
|  | AvgW1Fluids | Average fluids during the first week of life in ml/kg/day | x | - |
|  | AvgW1Glucose | Average glucose during the first week of life in ml/kg/day | x | - |
|  | AvgW1Lipids | Average lipids during the first week of life in ml/kg/day | x | -- |
|  | AvgW1Proteins | Average proteins during the first week of life in ml/kg/day |  | - |
|  | MaxCaloriesEN | Maximum enteral calories in kcal/kg/day |  | - |
|  | MaxNonProteinCaloriesEN | Maximum enteral non-protein caloriesin kcal/kg/day |  | - |
|  | MaxProteinCaloriesEN | Maximum enteral protein calories in kcal/kg/day |  | - |
|  | MaxFluidsEN | Maximum enteral fluids in ml/kg/day |  | - |
|  | MaxGlucoseEN | Maximum enteral glucose in ml/kg/day |  | - |
|  | MaxLipidsPN | Maximum enteral lipids in ml/kg/day |  | - |
|  | MaxProteinsEN | Maximum enteral proteins life in ml/kg/day | x | - |
|  | MaxCaloriesPN | Maximum parenteral calories in kcal/kg/day |  | - |
|  | MaxNonProteinCaloriesPN | Maximum parenteral non-protein caloriesin kcal/kg/day |  | - |
|  | MaxProteinCaloriesPN | Maximum parenteral protein calories in kcal/kg/day |  | - |
|  | MaxFluidsPN | Maximum parenteral fluids in ml/kg/day |  | - |
|  | MaxGlucosePN | Maximum parenteral glucose in ml/kg/day |  | - |
|  | MaxLipidsEN | Maximum parenteral lipids in ml/kg/day |  | - |
|  | MaxProteinsPN | Maximum parenteral proteins life in ml/kg/day |  | - |
|  | MaxCalories | Maximum calories in kcal/kg/day |  | - |
|  | MaxFluids | Maximum non-protein caloriesin kcal/kg/day |  | - |
|  | MaxGlucose | Maximum protein calories in kcal/kg/day |  | - |
|  | MaxLipids | Maximum fluids in ml/kg/day |  | - |
|  | MaxNonProteinCalories | Maximum glucose in ml/kg/day |  | - |
|  | MaxProteinCalories | Maximum lipids in ml/kg/day |  | - |
|  | MaxProteins | Maximum enteral proteins life in ml/kg/day |  | - |
|  | MinCaloriesEN | Minimum enteral calories in kcal/kg/day |  | - |
|  | MinNonProteinCaloriesEN | Minimum enteral non-protein caloriesin kcal/kg/day |  | - |
|  | MinProteinCaloriesEN | Minimum enteral protein calories in kcal/kg/day |  | - |
|  | MinFluidsEN | Minimum enteral fluids in ml/kg/day |  | - |
|  | MinGlucoseEN | Minimum enteral glucose in ml/kg/day |  | - |
|  | MinLipidsEN | Minimum enteral lipids in ml/kg/day |  | - |
|  | MinProteinsEN | Minimum enteral proteins life in ml/kg/day | x | - |
|  | MinCaloriesPN | Minimum parenteral calories in kcal/kg/day |  | - |
|  | MinNonProteinCaloriesPN | Minimum parenteral non-protein caloriesin kcal/kg/day | x | - |
|  | MinProteinCaloriesPN | Minimum parenteral protein calories in kcal/kg/day |  | - |
|  | MinFluidsPN | Minimum parenteral fluids in ml/kg/day |  | - |
|  | MinGlucosePN | Minimum parenteral glucose in ml/kg/day |  | - |
|  | MinLipidsPN | Minimum parenteral lipids in ml/kg/day | x | - |
|  | MinProteinsPN | Minimum parenteral proteins life in ml/kg/day | x | - |
|  | MinCalories | Minimum calories in kcal/kg/day |  | - |
|  | MinNonProteinCalories | Minimum non-protein caloriesin kcal/kg/day |  | - |
|  | MinProteinCalories | Minimum protein calories in kcal/kg/day |  | - |
|  | MinFluids | Minimum fluids in ml/kg/day |  | - |
|  | MinGlucose | Minimum glucose in ml/kg/day |  | - |
|  | MinLipids | Minimum lipids in ml/kg/day |  | - |
|  | MinProteins | Minimum enteral proteins life in ml/kg/day |  | - |
|  | DaysToMaxCaloriesEN | Number of days to reach the maximum pro kg enteral calories |  | - |
|  | DaysToMaxNonProteinCaloriesEN | Number of days to reach the maximum pro kg enteral non-protein calories |  | - |
|  | DaysToMaxProteinCaloriesEN | Number of days to reach the maximum pro kg enteral protein calories |  | - |
|  | DaysToMaxFluidsEN | Number of days to reach the maximum pro kg enteral fluids |  | - |
|  | DaysToMaxGlucoseEN | Number of days to reach the maximum pro kg enteral glucose amount |  | - |
|  | DaysToMaxLipidsEN | Number of days to reach the maximum pro kg enteral lipids amount |  | - |
|  | DaysToMaxProteinsEN | Number of days to reach the maximum pro kg enteral proteins amount |  | - |
|  | DaysToMaxCaloriesPN | Number of days to reach the maximum pro kg parenteral calories |  | - |
|  | DaysToMaxNonProteinCaloriesPN | Number of days to reach the maximum pro kg parenteral non-protein calories |  | - |
|  | DaysToMaxProteinCaloriesPN | Number of days to reach the maximum pro kg parenteral protein calories |  | - |
|  | DaysToMaxFluidsPN | Number of days to reach the maximum pro kg parenteral fluids |  | - |
|  | DaysToMaxGlucosePN | Number of days to reach the maximum pro kg parenteral glucose amount |  | - |
|  | DaysToMaxLipidsPN | Number of days to reach the maximum pro kg parenteral lipids amount |  | - |
|  | DaysToMaxProteinsPN | Number of days to reach the maximum pro kg parenteral proteins amount |  | - |
|  | DaysToMaxCalories | Number of days to reach the maximum pro kg calories |  | - |
|  | DaysToMaxFluids | Number of days to reach the maximum pro kg non-protein calories |  | - |
|  | DaysToMaxGlucose | Number of days to reach the maximum pro kg protein calories |  | - |
|  | DaysToMaxLipids | Number of days to reach the maximum pro kg fluids |  | - |
|  | DaysToMaxNonProteinCalories | Number of days to reach the maximum pro kg glucose amount |  | - |
|  | DaysToMaxProteinCalories | Number of days to reach the maximum pro kg lipids amount |  | - |
|  | DaysToMaxProteins | Number of days to reach the maximum pro kg proteins amount |  | - |
|  | DaysPNlipids | Number of days with parenteral lipids |  | - |
|  | ENcaloriesStopPN | Enteral calories on the first day of FEF | x | - |
| Ventilation | InvasiveVentilation | Proportion of hours from birth until the end of TN with invasive respiratory support over hours from birth to FEF | x | - |
|  | NitricOxide | Proportion of hours from birth until the end of TN with nitric oxide respiratory support over hours from birth to FEF | x | - |
|  | NonInvasiveVentilation | Proportion of hours from birth until the end of TN with non-invasive respiratory support over hours from birth to FEF | x | - |
| Complications | OxyDep36w | Oxygen dependency at 36 weeks postmentrual age | x | - |
|  | OxyDep28d | Oxygen dependency at 28 days of life | x | - |
|  | NEC | Necrotizing enterocolitis | x | - |
|  | NEC_IP | Necrotizing enterocolitis with intestinal perforation | x | - |
|  | EarlyOnsetSepsis | Sepsis identified from antibiotic therapy of at least 5 consecutive days, starting within 72 hours from birth | x | - |
|  | LateOnsetSepsis | Sepsis identified from antibiotic therapy of at least 5 consecutive days, starting later than 72 hours from birth | x | - |
| Antibiotics | CumulativeAntibiotic | Overall amount of administered antibiotics in mg/kg | x | - |
|  | AvgAntibiotic | Average amount of antibiotics from birth to the end of TN phase in mg/kg/day | x | - |
| Growth | GV1w | Average growth velocity during the first week of life in g/kg/day | x | - |

*eTable 1: Features list*

*
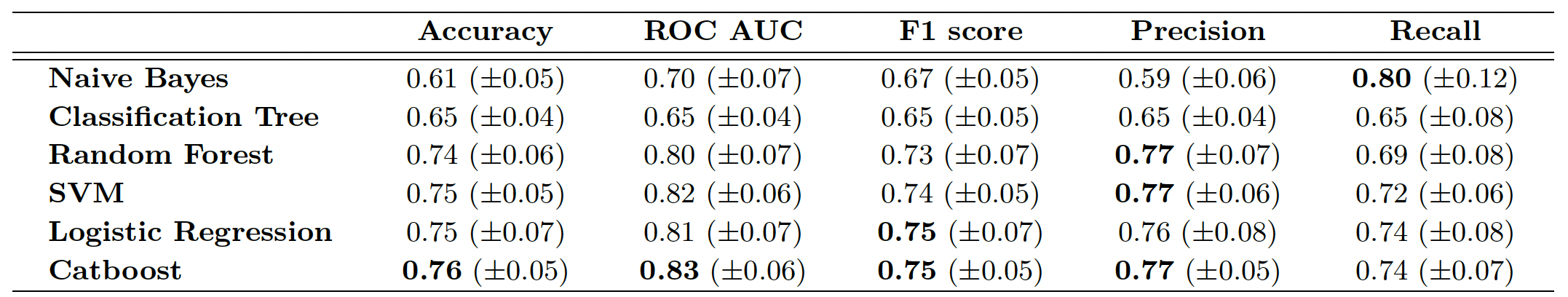
*

*eTable 2: Classification algorithm choice. Mean (± standard deviation) for 10-fold cross-validation. The best result in bold.*

|  | **All features** | | **Nutritional features** | |
| --- | --- | --- | --- | --- |
|  | **Cross-validation** | **External validation** | **Cross-validation** | **External validation** |
| *All patients* | 0.76 ± 0.05 | 0.77 | 0.68 ± 0.07 | 0.70 |
| *Moderate-to-late preterm* | 0.74 ± 0.06 | 0.77 | 0.68 ± 0.05 | 0.71 |
| *Very preterm* | 0.73 ± 0.09 | 0.75 | 0.65 ± 0.07 | 0.69 |
| *Extremely preterm* | 0.74 ± 0.05 | 0.74 | 0.63 ± 0.09 | 0.68 |
| *SGA* | 0.71 ± 0.05 | 0.70 | 0.70 ± 0.02 | 0.65 |

*eTable 3: Cross-validation (i.e., internal validation) and external validation accuracy results from one random split of the dataset.*
